# One-Year Gaps in Comprehensive Secondary Prevention After Acute Myocardial Infarction: Statin Persistence, LDL-C Target Achievement, Rehabilitation, and Lifestyle Adherence

**DOI:** 10.3390/medicina62071342

**Published:** 2026-07-12

**Authors:** Anđela Jurišić, Ivana Jurin, Marin Pavlov, Šime Manola, Antonio Patrk, Anica Gavran, Boris Starčević, Irzal Hadžibegović, Igor Rudež

**Affiliations:** 1 Department of Cardiology, Dubrava University Hospital, 10000 Zagreb, Croatia; andjelajurisic1@gmail.com (A.J.); sime.manola@icloud.com (Š.M.); irzalh@gmail.com (I.H.); 2 University Centre Koprivnica, University North, 48000 Koprivnica, Croatia; 3 School of Medicine, University of Zagreb, 10000 Zagreb, Croatia; 4 Department of Cardiology, University Hospital Centre Zagreb, 10000 Zagreb, Croatia; anica.milinkovic18@gmail.com; 5 Department of Cardiology, Merkur University Hospital, 10000 Zagreb, Croatia; 6 Faculty of Dental Medicine and Health Osijek, Josip Juraj Strossmayer University of Osijek, 31000 Osijek, Croatia; 7 Department of Cardiac Surgery, Dubrava University Hospital, 10000 Zagreb, Croatia

**Keywords:** acute myocardial infarction, secondary prevention, LDL-C target, statin persistence, cardiac rehabilitation, lifestyle, smoking cessation, registry

## Abstract

*Background and Objectives*: Secondary prevention after acute myocardial infarction (AMI) is often assessed by discharge prescribing, yet first-year prognosis depends on whether pharmacological, rehabilitation and lifestyle measures are completed after discharge. We evaluated one-year secondary prevention pathway completion after AMI and its relationship with LDL-C target achievement and exploratory outcomes. *Materials and Methods*: Consecutive STEMI/NSTEMI patients with documented follow-up were identified from the Cardiology Research Dubrava registry. We assessed discharge lipid-lowering therapy, statin persistence, LDL-C <1.4 mmol/L at 12 months, cardiac rehabilitation, exercise, dietary pattern, smoking status, left ventricular ejection fraction, angiographic complexity, biomarkers and one-year outcomes. *Results*: Among 2976 patients, statins were prescribed at discharge in 2782/2838 (98.0%), but LDL-C <1.4 mmol/L was achieved in 749/2566 (29.2%). Statin discontinuation or irregular use occurred in 915/2619 (34.9%) and was strongly associated with failure to reach target. Rehabilitation, exercise, favorable dietary pattern and smoke-free status were incompletely achieved and clustered with better LDL-C target attainment. One-year all-cause mortality was 288/2942 (9.8%), and ischemic MACE occurred in 395/2902 (13.6%). Events were associated with older age, reduced LVEF, greater angiographic complexity and higher inflammatory/neurohormonal burden. *Conclusions*: After AMI, the main first-year prevention gap was not discharge statin prescribing but post-discharge pathway completion. These findings support structured follow-up focused on statin persistence, LDL-C monitoring, treatment intensification, rehabilitation and lifestyle domains.

## 1. Introduction

Acute myocardial infarction (AMI) remains a major source of early and late cardiovascular morbidity despite major advances in reperfusion therapy, antithrombotic treatment and evidence-based pharmacological care. Contemporary AMI management is therefore not complete at the time of successful coronary angiography, percutaneous coronary intervention or discharge prescription. The first year after AMI is a vulnerable period during which residual risk is shaped by whether guideline-directed secondary-prevention measures are initiated, sustained, monitored and intensified in routine care [1].

Secondary prevention after AMI is multidimensional. Lipid-lowering therapy is a central component: European dyslipidemia guidelines consider post-AMI patients to be at very high risk and recommend low-density lipoprotein cholesterol (LDL-C) <1.4 mmol/L together with at least a 50% reduction from baseline [2]. Broader cardiovascular prevention guidance also emphasizes smoking cessation, physical activity, weight and metabolic risk management, healthy dietary patterns and structured cardiac rehabilitation as core elements of long-term risk reduction [3,4]. These domains are clinically linked, but in everyday practice they are often delivered by different teams, assessed at different time points and incompletely documented.

Large registries have consistently shown that implementation remains incomplete. EUROASPIRE V demonstrated persistent lifestyle and risk-factor gaps among coronary patients across Europe, while the SANTORINI study showed that LDL-C goal attainment remains suboptimal even among high- and very-high-risk patients treated in contemporary European practice [5,6]. The SWEDEHEART secondary-prevention and cardiac-rehabilitation registry has shown the value of structured follow-up after myocardial infarction [7]. In parallel, observational data indicate that actual statin adherence and achievement of lipid targets are closely related to outcomes after myocardial infarction [8]. FAST-MI analyses have supported the prognostic relevance of rehabilitation referral in selected post-AMI populations [9].

However, much of the existing literature evaluates individual prevention domains—lipid target achievement, rehabilitation attendance, smoking cessation, dietary behavior, exercise, or clinical outcomes—separately. This approach may underestimate the practical problem faced by clinicians and patients: secondary prevention is a pathway rather than an isolated intervention. Discharge prescribing may be excellent, while post-discharge persistence, follow-up lipid testing, therapy escalation, rehabilitation participation and lifestyle adherence remain incomplete. Furthermore, the relationship between completion of the prevention process and clinical outcomes must be interpreted together with baseline disease severity, left ventricular function, angiographic complexity and inflammatory or neurohormonal burden.

The present study therefore aimed to describe first-year secondary-prevention pathway completion after AMI in a real-world patient-level registry. We focused on discharge statin prescribing, statin persistence, LDL-C target achievement, cardiac rehabilitation, exercise, observed dietary pattern and smoking status, and examined how these domains related to sex-specific profiles, baseline clinical severity, biomarkers and exploratory one-year outcomes.

## 2. Materials and Methods

### 2.1. Study Design, Population and Ethics

Patients were identified from the Cardiology Research Dubrava (CaRD) registry (ClinicalTrials.gov identifier: NCT06090591; registry protocol DUH-012017), a prospectively maintained all-comer cardiovascular registry of a tertiary cardiovascular center. The registry captures clinical presentation, cardiovascular risk factors, laboratory values, echocardiographic findings, angiographic and procedural characteristics, discharge therapy and follow-up data from routine care. The present analysis included consecutive adult patients hospitalized from January 2017 onward with definite ST-elevation myocardial infarction (STEMI) or non-ST-elevation myocardial infarction (NSTEMI), as defined by contemporary guidelines and the Fourth Universal Definition of Myocardial Infarction, who underwent coronary angiography during the index hospitalization [1,10].

Patients with unstable angina, unclear acute coronary syndrome subtype, an alternative final diagnosis after diagnostic work-up, missing follow-up information or invalid follow-up time were not included in the primary AMI follow-up cohort. Patients initially suspected of AMI but later diagnosed with Takotsubo cardiomyopathy, pericarditis, myocarditis or another non-coronary condition mimicking AMI were excluded. Patients with AMI and no obstructive coronary artery disease were retained as myocardial infarction with non-obstructive coronary arteries (MINOCA) and described separately because their secondary prevention pathway may differ from obstructive coronary disease.

Analyses were performed at the patient level. The index AMI hospitalization was used as the anchor for follow-up and for all baseline clinical, laboratory, echocardiographic and angiographic variables. When more than one registry record referred to the same patient, data from the index hospitalization were linked to the most complete documented follow-up record. Follow-up status was determined from outpatient visits, rehospitalization records, and telephone contact. For one-year outcome analyses, patients were considered evaluable if an event occurred within 365 days after AMI or if at least 365 event-free days were documented. In-hospital deaths were retained as one-year events when timing and cause of death were documented.

The study was conducted in accordance with the Declaration of Helsinki and approved by the Ethics Committee of Dubrava University Hospital (protocol code 2020/2409-08, approval date: 24 September 2020). Written informed consent was obtained from all subjects involved in the CaRD registry. The consent covered participation in scientific research and permission for access to relevant medical documentation by the responsible study personnel and ethics committee representatives. Lifestyle, exercise and dietary questionnaire items analyzed in the present manuscript were collected as part of routine CaRD registry follow-up and are reported only as de-identified aggregate results.

### 2.2. Clinical, Laboratory, Echocardiographic and Angiographic Variables

Demographic, clinical and laboratory data were recorded during the index hospitalization. Cardiovascular risk factors and previous cardiovascular disease were abstracted from medical documentation, including hypertension, diabetes mellitus, hyperlipidemia, active smoking at presentation, prior myocardial infarction, prior percutaneous coronary intervention and prior coronary artery bypass grafting. Anthropometric variables included body mass index (BMI) when available. Glycemic information included diabetes status and glycated hemoglobin (HbA1c) values when recorded during routine care.

Routine laboratory data included admission lipid profile and, when available, C-reactive protein (CRP), fibrinogen, albumin, neutrophil-to-lymphocyte ratio and N-terminal pro-B-type natriuretic peptide (NT-proBNP). These biomarkers were used to characterize inflammatory, nutritional and neurohormonal burden rather than to define the prevention process. Left ventricular ejection fraction (LVEF) was assessed by transthoracic echocardiography during the index hospitalization or before discharge when available.

All patients underwent coronary angiography during the index hospitalization. Culprit vessel, multivessel disease, chronic total occlusion and revascularization strategy were recorded from angiographic and procedural reports. When a culprit lesion was confirmed, percutaneous coronary intervention was performed according to operator judgment. In patients with multivessel disease, the decision for ad hoc complete revascularization, staged percutaneous coronary intervention or coronary artery bypass grafting was made by the operator or by the institutional heart team. Angiographic complexity was assessed by the SYNTAX (Synergy between Percutaneous Coronary Intervention with TAXUS and Cardiac Surgery) score when available [11].

### 2.3. Pharmacological Secondary Prevention and LDL-C Target Achievement

Medications at discharge were abstracted from discharge documentation. Statin prescription, high-intensity statin therapy and ezetimibe-containing regimens were recorded. LDL-C values were retrieved from routine clinical laboratory measurements during the index hospitalization and at follow-up. The primary lipid endpoint was LDL-C <1.4 mmol/L at 12 months, reflecting current very-high-risk secondary prevention recommendations [2].

After discharge, patients were followed through routine outpatient visits and telephone contact. These contacts were used to record medication use, adverse effects, dose changes and events not captured during routine visits. First-year statin non-persistence or irregular use was defined as documented discontinuation of statin therapy or irregular use during the first year after AMI. When available, the recorded reason for discontinuation or irregular use was categorized into adverse effects, patients’ belief that lipid levels were already controlled, forgetfulness or uncertainty, perceived lack of need, patient report of taking therapy despite registry-classified irregularity, physician-related change, cost or access issues, and other or unclear reasons. Follow-up treatment intensification with ezetimibe, proprotein convertase subtilisin/kexin type 9 (PCSK9) inhibitors, inclisiran or bempedoic acid was summarized descriptively because these variables were not captured as uniform prespecified endpoints throughout the entire registry period.

### 2.4. Rehabilitation, Lifestyle Domains and Process-of-Care Measure

Participation in cardiac rehabilitation was recorded when patients attended a structured post-AMI rehabilitation program. Regular exercise was defined as patient-reported moderate physical activity for at least 30 min on 5–7 days per week according to the registry questionnaire. Smoking status was recorded at the index AMI hospitalization and at follow-up. Smoking cessation was evaluated only among patients who were active smokers at the index event and had follow-up smoking data.

Dietary information was analyzed only when recorded during follow-up. Dietary habits were assessed with a structured food-frequency questionnaire module used in routine registry follow-up. The module recorded predefined food groups relevant to cardiovascular prevention, including vegetables or salad, fruit, fish, whole-grain bread, olive oil, white bread, sweets or pastries, butter or lard, and processed meat products. The item-based approach was aligned with short food-frequency and Mediterranean diet screening instruments previously used in cardiovascular risk and secondary prevention populations, including the PREDIMED Mediterranean Diet Adherence Screener and the CORDIOPREV secondary prevention dietary trial [12,13,14]. A favorable dietary pattern was defined a priori as at least three favorable components and no more than one unfavorable daily component. Favorable components included daily salad or vegetables, daily fruit, any fish intake, any olive oil use and daily whole-grain bread. Unfavorable components included daily processed meat, white bread, sweets, butter or lard. For the graded display, an observed dietary pattern score was calculated by summing the five favorable components (range 0–5) and was grouped as low (≤1 component), intermediate (2–3 components) and high (≥4 components). This classification was used as a descriptive dietary pattern measure and was not interpreted as a formally validated Mediterranean diet score in this AMI cohort.

A non-statin process-of-care measure was constructed for descriptive analyses of pathway completion. Because statin persistence is a direct mechanistic determinant of LDL-C target achievement, it was not included in the process measure used for LDL-C analyses. One point was assigned for each non-statin domain: cardiac rehabilitation, regular exercise, favorable dietary pattern and smoke-free follow-up status. Completion was categorized as low (0–1 points), intermediate (2–3 points), or high (4 points). This measure was designed as a pragmatic process-of-care descriptor, not as a validated risk score. Lifestyle and dietary variables were analyzed as observed data only; missing values were not imputed.

### 2.5. One-Year Outcomes

Clinical outcomes were assessed within 365 days after the index AMI. Patients were evaluable for a given one-year outcome if the event occurred within 365 days or if at least 365 event-free days were documented. The main descriptive clinical outcomes were all-cause death, cardiovascular death, ischemic major adverse cardiovascular events (MACE) and broad MACE. Outcomes were derived from hospitalization records, outpatient documentation, death information and telephone follow-up.

Cardiovascular death was defined as death due to sudden death, myocardial infarction, heart failure, stroke or pulmonary embolism. Ischemic MACE included cardiovascular death, recurrent myocardial infarction, stroke or transient ischemic attack, unplanned coronary revascularization, stent or bypass-graft occlusion or restenosis and stent thrombosis. Broad MACE additionally included heart failure events, major bleeding, pulmonary embolism or venous thromboembolism and other clearly coded cardiovascular events. Because events were registry-based and not adjudicated by an independent clinical events committee, all MACE analyses were considered exploratory.

### 2.6. Statistical Analysis

Continuous variables are presented as median [interquartile range] and categorical variables as n/N (%). Between-group comparisons used non-parametric tests for continuous variables and chi-square or Fisher’s exact tests for categorical variables, as appropriate. Sex-specific comparisons are reported with *p*-values. Missing data were not imputed; denominators are shown for each variable, and analyses involving lifestyle, diet or smoking were restricted to patients with available observed data. Patients with and without complete lifestyle data were compared to characterize potential informative missingness.

Adjusted logistic regression was used to explore associations with LDL-C target achievement and one-year outcomes. The LDL-C target model included statin non-persistence, ezetimibe-containing discharge therapy, admission LDL-C, the non-statin process-of-care measure, age, sex and diabetes. One-year outcome models included baseline and acute-severity variables available before or during the index hospitalization: age, sex, diabetes, prior myocardial infarction, STEMI presentation, LVEF <40%, SYNTAX score and CRP above the median. Post-discharge rehabilitation and lifestyle variables were not included in one-year mortality or MACE models because they are susceptible to immortal-time, survivor and healthy-user bias. Results are presented as odds ratios with 95% confidence intervals. Analyses were performed in Python 3.11. Reporting followed the Strengthening the Reporting of Observational Studies in Epidemiology (STROBE) recommendations [15].

## 3. Results

### 3.1. Cohort and One-Year Outcomes

**Figure 1:**
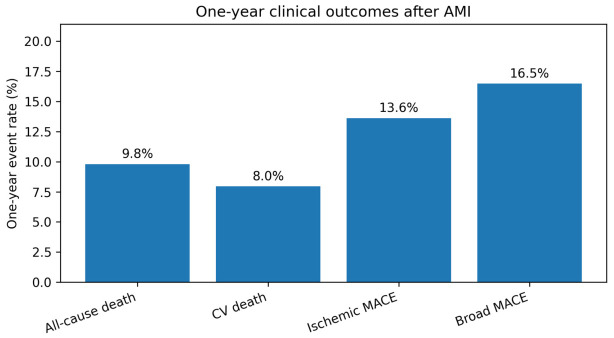
One-year clinical outcomes after acute myocardial infarction. CV, cardiovascular; MACE, major adverse cardiovascular events.

The final cohort included 2976 AMI patients with documented follow-up: 1648 (55.4%) with STEMI and 1328 (44.6%) with NSTEMI. Median follow-up was 1140 days (interquartile range 412–1854). One-year outcomes were evaluable in 2942/2976 (98.9%) patients for mortality and 2902/2976 (97.5%) patients for MACE. One-year all-cause mortality was 288/2942 (9.8%), cardiovascular mortality 234/2942 (8.0%), ischemic MACE 395/2902 (13.6%) and broad MACE 478/2902 (16.5%) (Figure 1). MINOCA was uncommon (6/2976; 0.2%) and is described in the Supplementary Materials. Thus, the cohort predominantly represented angiographically confirmed STEMI or NSTEMI treated in a tertiary invasive-care setting.

### 3.2. Sex-Specific Secondary Prevention Profile

Women were older than men and had a higher prevalence of hypertension, whereas active smoking at presentation was more common in men. Women had lower rates of participation in cardiac rehabilitation and regular exercise. LDL-C target achievement was numerically lower in women, but the difference was of borderline statistical significance. One-year ischemic and broad MACE did not differ significantly by sex, whereas one-year mortality was higher in women (Table 1). These findings suggest that sex-specific secondary prevention gaps were not confined to pharmacological treatment but also involved rehabilitation and lifestyle engagement.

### 3.3. Statin Persistence, LDL-C Target Achievement and Treatment Intensification

Statins were prescribed at discharge in 2782/2838 (98.0%) patients, and high-intensity statin or combination therapy was used in 2630/2780 (94.6%). Nevertheless, LDL-C <1.4 mmol/L at 12 months was achieved in only 749/2566 (29.2%). Statin discontinuation or irregular use during the first year occurred in 915/2619 (34.9%). LDL-C target achievement was 698/1659 (42.1%) among persistent statin users compared with 41/870 (4.7%) among patients with discontinued or irregular statin use, identifying persistence as the clearest modifiable pharmacological gap (Table 2).

Data on reasons were available for 713 patients with statin discontinuation or irregular use and revealed heterogeneous categories rather than a single dominant mechanism. The most frequently recorded categories were the perception that lipid levels were adequate (189/713; 26.5%), patient-reported use of therapy despite registry-classified irregularity (167/713; 23.4%), forgetfulness or uncertainty (114/713; 16.0%), adverse effects as the primary recorded reason (73/713; 10.2%), and perceived lack of need (65/713; 9.1%). Any reported statin side effect, irrespective of whether it was assigned as the primary reason for non-persistence or irregular use, was documented in 179/714 (25.1%) non-persistent or irregular users. Ezetimibe-containing therapy was prescribed at discharge in 389/2780 (14.0%). Follow-up intensification was incompletely captured; descriptively, use of or addition of an ezetimibe-containing regimen was recorded in 548/2976 (18.4%) overall and in 419/1817 (23.1%) LDL-C non-target patients, while use, addition, or recommendation of a PCSK9 inhibitor, inclisiran, or bempedoic acid was recorded in 59/2976 (2.0%) overall and in 40/1817 (2.2%) LDL-C non-target patients (Supplementary Tables S4 and S5). These data indicate that low target achievement reflected both adherence/persistence problems and incomplete treatment intensification during follow-up.

### 3.4. Rehabilitation, Lifestyle Domains, Dietary Patterns and Missing Data

**Figure 2:**
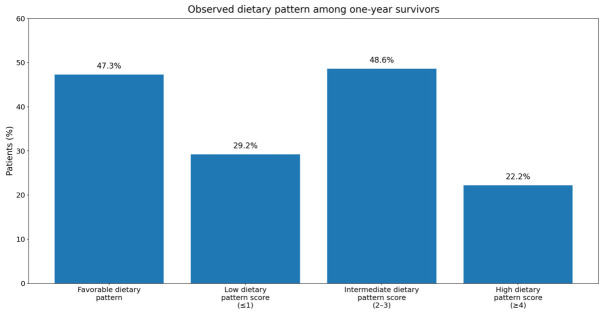
Observed dietary pattern in patients with available follow-up dietary data. Dietary habits were assessed using structured food-frequency items. The definition of a favorable dietary pattern was pragmatic and descriptive and was not interpreted as a formally validated Mediterranean diet score in this AMI cohort.

Cardiac rehabilitation was recorded in 1396/2688 (51.9%) patients, regular exercise in 761/2028 (37.5%) and favorable dietary pattern in 1046/2210 (47.3%) patients with observed dietary data (Figure 2). Active smoking at AMI was recorded in 1465/2968 (49.4%) patients with available baseline smoking data, while active smoking at follow-up was recorded in 585/2046 (28.6%) patients with available follow-up smoking data. Because smoking variables were derived from available baseline and paired follow-up data, denominators differ across baseline smoking, follow-up smoking and smoking cessation analyses. Among baseline smokers with follow-up smoking data, 500/1044 (47.9%) stopped smoking, while 544/1044 (52.1%) continued smoking. Smoking cessation was more frequent among patients participating in rehabilitation than among those not participating: 327/636 (51.4%) vs. 172/405 (42.5%).

Complete observed data for exercise, dietary pattern and follow-up smoking status were available in 2002/2976 (67.3%) patients. Patients without complete lifestyle data were older, more often women, more often diabetic and had lower LVEF, higher SYNTAX score and higher one-year event rates (Supplementary Table S2). Therefore, incomplete lifestyle ascertainment was considered potentially informative rather than missing completely at random. Anthropometric and glycemic data are summarized in Supplementary Table S6: median baseline BMI was 28.4 kg/m^2^, obesity was present in 1063/2853 (37.3%), paired BMI change was small (median −0.2 kg/m^2^) and follow-up HbA1c and contemporary glucose-lowering therapy data were available only in subsets.

**Figure 3:**
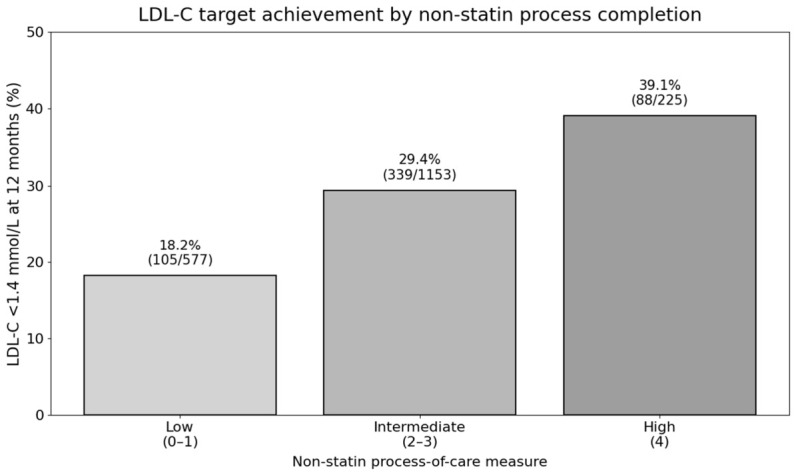
LDL-C target achievement according to a non-statin process-of-care measure that included rehabilitation, regular exercise, favorable dietary pattern and smoke-free follow-up status. Statin persistence was intentionally excluded from this measure to avoid circularity when analyzing LDL-C target achievement. LDL-C, low-density lipoprotein cholesterol.

When statin persistence was excluded from the process-of-care measure to avoid circularity with LDL-C target achievement, LDL-C <1.4 mmol/L increased across non-statin pathway completion categories: 105/577 (18.2%) in the low group, 339/1153 (29.4%) in the intermediate group and 88/225 (39.1%) in the high group (Figure 3). This gradient should be interpreted as descriptive co-occurrence between observed non-statin prevention domains and LDL-C target achievement, not as evidence that the composite independently explains lipid control. In the adjusted LDL-C target model, statin discontinuation or irregular use remained the dominant determinant of target failure, while the non-statin process-of-care measure remained independently associated with LDL-C target achievement (Table 3 and Supplementary Table S7).

### 3.5. Baseline Severity, Biomarkers and Exploratory One-Year Outcome Models

One-year mortality and MACE were associated with older age, reduced LVEF, greater angiographic complexity and higher inflammatory or neurohormonal burden. Patients with one-year events had higher CRP, fibrinogen, neutrophil-to-lymphocyte ratio and NT-proBNP and lower albumin (Supplementary Table S9). These biomarkers were not used to define the prevention pathway, but they help characterize the baseline and acute-risk phenotype of patients who experienced early adverse outcomes.

In exploratory models adjusted for baseline characteristics and disease severity, post-discharge rehabilitation and follow-up lifestyle variables were excluded; one-year mortality was associated with older age, LVEF <40%, higher SYNTAX score and CRP above the median. Similar patterns were observed for ischemic and broad MACE (Table 3 and Supplementary Table S8). Because MACE events were registry-based and not independently adjudicated, these models were used to contextualize baseline severity rather than to establish causality or provide definitive outcome predictions.

## 4. Discussion

The principal finding of this study is that the first-year secondary-prevention gap after AMI occurred predominantly after discharge. Statin prescribing at discharge was almost universal, and high-intensity or combination lipid-lowering therapy was frequent, yet fewer than one-third of patients reached LDL-C <1.4 mmol/L at 12 months. The clearest pharmacological failure point was statin discontinuation or irregular use. In parallel, cardiac rehabilitation, regular exercise, favorable dietary pattern and smoking cessation were incompletely achieved, indicating that early post-AMI prevention is best understood as a pathway problem rather than a prescription problem.

These findings are consistent with, but add to, prior European observations. EUROASPIRE V and SANTORINI established that risk-factor control and LDL-C goal attainment remain suboptimal in coronary and very-high-risk patients [5,6]. The SWEDEHEART-CR registry and FAST-MI analyses demonstrated the clinical relevance of structured secondary prevention and rehabilitation [7,9]. The present CaRD registry analysis adds patient-level integration of these domains within one first-year AMI framework, linking discharge therapy, medication persistence, LDL-C target achievement, rehabilitation, observed lifestyle domains, ventricular function, angiographic complexity, biomarkers and one-year events. This study therefore identifies not only whether prevention targets were missed but also where the post-discharge pathway appeared to fragment.

Statin non-persistence was the strongest modifiable pharmacological failure point. Patients with discontinued or irregular statin use rarely achieved the LDL-C target. Importantly, documented reasons for non-persistence were heterogeneous. Adverse effects were recorded in only a subset, while beliefs that lipid levels were adequate, uncertainty and forgetfulness were also frequent. This distinction matters clinically because true statin intolerance, low perceived need and simple non-adherence require different interventions. The results support systematic follow-up of actual medication use, not only discharge prescriptions [16].

The low proportion of patients achieving LDL-C targets also raises the question of therapeutic escalation. Ezetimibe use at discharge was modest, and recorded follow-up intensification to ezetimibe, PCSK9 inhibitors, inclisiran or bempedoic acid was infrequent, including among patients who remained above the LDL-C target. These data were not captured as a uniform prespecified endpoint throughout the study period and should therefore be interpreted descriptively. Nevertheless, they suggest that failure to intensify lipid-lowering therapy may have contributed to residual LDL-C elevation. Contemporary evidence supports additional LDL-C lowering in very-high-risk patients who remain above target despite statin therapy [17,18].

Rehabilitation and lifestyle findings require careful interpretation. Cardiac rehabilitation participation, regular exercise, dietary pattern and smoke-free status clustered with better LDL-C target achievement, but these associations should not be interpreted as causal proof. Rehabilitation participation is strongly susceptible to healthy-user, referral and survivor bias. For this reason, rehabilitation was not included as an exposure in one-year mortality or MACE models. Instead, we interpret rehabilitation and lifestyle variables as indicators of structured prevention engagement and care pathway completion [14,16,19,20].

To avoid circularity between statin persistence and LDL-C target achievement, the process-of-care measure used in LDL-C analyses intentionally excluded statin persistence and included only rehabilitation, regular exercise, favorable dietary pattern and smoke-free follow-up status. Its graded relationship with LDL-C target achievement should be viewed as a descriptive co-occurrence of prevention behaviors and follow-up engagement, not as a validated risk score or causal mechanism. Equal weighting of these domains was pragmatic and should not be interpreted as implying equal biological importance. Statin persistence remained independently and strongly associated with LDL-C target achievement when analyzed separately.

The clinical implication is that quality assessment after AMI should not stop at discharge prescribing. A patient discharged on a high-intensity statin may still fail secondary prevention if medication use is not sustained, LDL-C is not rechecked, therapy is not intensified when targets are missed, rehabilitation is not completed, smoking persists, exercise remains low and dietary patterns do not improve. These domains are usually assessed in different settings, but the patient experiences them as one care pathway. Practical applications of this work include structured first-year follow-up checklists, early LDL-C reassessment, explicit documentation of statin intolerance versus non-adherence, automatic referral and attendance tracking for rehabilitation, and routine recording of smoking, exercise and dietary domains.

Future research should test whether pathway-based secondary prevention interventions improve both risk-factor control and clinical outcomes after AMI. Such studies should ideally use multicenter, prospective designs and include standardized lifestyle instruments, adjudicated endpoints, repeated LDL-C measurements, explicit treatment escalation algorithms and patient-reported barriers to medication persistence and rehabilitation participation. Digital follow-up tools, pharmacy-linked adherence data and rehabilitation referral-to-attendance tracking may help distinguish non-adherence, therapeutic inertia, access barriers and clinical contraindications. The non-statin process measure used here should therefore be considered hypothesis-generating and should undergo external validation before clinical use.

The study also identifies important limitations in real-world data capture. Lifestyle variables were not available in all participants, and patients without complete observed lifestyle data appeared older, sicker and at higher risk. Missingness may therefore reflect fragmented follow-up or early clinical deterioration rather than random data loss. Similarly, regular exercise was assessed by a registry questionnaire, and dietary pattern was derived from structured food-frequency items but was not formally validated as a Mediterranean diet score in this AMI cohort. Baseline BMI and obesity were available in most patients, but follow-up BMI, HbA1c and contemporary glucose-lowering therapy data were incomplete, limiting inference about weight change and cardiometabolic treatment. These limitations are important, but they also mirror the practical challenges of evaluating secondary prevention in routine care.

Several additional limitations should be acknowledged. This was a single-center observational registry analysis within the CaRD registry, and residual confounding is unavoidable. MACE analyses were exploratory because events were registry-based and were not adjudicated by an independent clinical events committee. The process-of-care measure was unvalidated, equally weighted and intended only to summarize observed care processes. Lifestyle variables were self-reported and available only in subsets. Anthropometric and glycemic data were incomplete, and contemporary cardiometabolic therapies were not captured uniformly; these variables may influence both pathway completion and outcomes. Only a small number of MINOCA patients were retained, limiting subgroup interpretation. Finally, although median follow-up exceeded one year, the present analysis focused on 365-day outcomes to match the first-year prevention pathway and reduce heterogeneity in follow-up duration.

## 5. Conclusions

In this real-world AMI cohort, first-year secondary prevention gaps occurred mainly after discharge. Statin prescribing was nearly universal, but LDL-C target achievement remained low and was strongly linked to actual statin persistence. Rehabilitation, exercise, dietary pattern and smoking cessation were incompletely achieved and were associated with lipid target attainment. One-year mortality and MACE reflected baseline clinical severity, ventricular function, angiographic complexity and inflammatory burden. These findings support a pathway-based approach to post-AMI care in which medication persistence, lipid monitoring, treatment intensification, rehabilitation and lifestyle domains are assessed together during the first year after infarction.

## Figures and Tables

**Table 1:** Sex-specific secondary prevention and one-year outcome profile. Values are median [interquartile range] or n/N (%). AMI, acute myocardial infarction; BMI, body mass index; LDL-C, low-density lipoprotein cholesterol; LVEF, left ventricular ejection fraction; MACE, major adverse cardiovascular events; STEMI, ST-elevation myocardial infarction; SYNTAX, Synergy between Percutaneous Coronary Intervention with TAXUS and Cardiac Surgery.

Variable	Overall	Men	Women	*p*
Age, years	64.0 [56.2–73.0]	62.0 [55.0–71.0]	69.0 [61.0–78.0]	<0.001
ST-elevation myocardial infarction	1648/2976 (55.4)	1169/2054 (56.9)	479/922 (52.0)	0.013
Diabetes mellitus	728/2976 (24.5)	484/2054 (23.6)	244/922 (26.5)	0.098
Hypertension	2209/2976 (74.2)	1464/2054 (71.3)	745/922 (80.8)	<0.001
Body mass index, kg/m^2^	28.4 [25.6–31.4]	28.6 [26.0–31.5]	28.0 [25.0–31.2]	0.001
Obesity, BMI ≥30 kg/m^2^	1063/2853 (37.3)	749/1967 (38.1)	314/886 (35.4)	0.191
Rehabilitation	1396/2688 (51.9)	1044/1872 (55.8)	352/816 (43.1)	<0.001
Regular exercise	761/2028 (37.5)	626/1438 (43.5)	135/590 (22.9)	<0.001
Favorable dietary pattern	1046/2210 (47.3)	732/1563 (46.8)	314/647 (48.5)	0.496
Active smoking at AMI	1465/2968 (49.4)	1114/2051 (54.3)	351/917 (38.3)	<0.001
Active smoking at follow-up	585/2046 (28.6)	453/1455 (31.1)	132/591 (22.3)	<0.001
LDL-C <1.4 mmol/L at 12 months	749/2566 (29.2)	549/1809 (30.3)	200/757 (26.4)	0.051
LVEF, %	52.0 [45.0–60.0]	51.0 [45.0–60.0]	54.0 [45.0–60.0]	0.009
LVEF <40%	370/2771 (13.4)	255/1913 (13.3)	115/858 (13.4)	1.000
SYNTAX score	14.5 [8.0–22.0]	15.0 [9.0–22.5]	12.5 [7.0–21.1]	<0.001
SYNTAX score ≥22	758/2911 (26.0)	553/2007 (27.6)	205/904 (22.7)	0.006
One-year death	288/2942 (9.8)	178/2035 (8.7)	110/907 (12.1)	0.005
One-year ischemic MACE	395/2902 (13.6)	264/2007 (13.2)	131/895 (14.6)	0.309
One-year broad MACE	478/2902 (16.5)	316/2007 (15.7)	162/895 (18.1)	0.127

**Table 2:** Secondary prevention pathway completion and LDL-C target achievement. LDL-C, low-density lipoprotein cholesterol.

Measure	Result
Statin prescription at discharge	2782/2838 (98.0)
High-intensity statin or combination regimen	2630/2780 (94.6)
Ezetimibe-containing discharge regimen	389/2780 (14.0)
Statin discontinued or irregular use during first year	915/2619 (34.9)
LDL-C <1.4 mmol/L at 12 months, persistent statin users	698/1659 (42.1)
LDL-C <1.4 mmol/L at 12 months, discontinued/irregular users	41/870 (4.7)
Favorable dietary pattern, observed data	1046/2210 (47.3)
Smoking cessation among baseline smokers	500/1044 (47.9)
LDL-C <1.4 mmol/L, non-statin process measure low 0–1	105/577 (18.2)
LDL-C <1.4 mmol/L, non-statin process measure intermediate 2–3	339/1153 (29.4)
LDL-C <1.4 mmol/L, non-statin process measure high 4	88/225 (39.1)

**Table 3:** Selected adjusted logistic regression models. LDL-C target model includes pharmacological and observed process variables. One-year outcome models include baseline and acute-severity variables only and are exploratory. CI, confidence interval; CRP, C-reactive protein; LDL-C, low-density lipoprotein cholesterol; LVEF, left ventricular ejection fraction; MACE, major adverse cardiovascular events; OR, odds ratio; SYNTAX, Synergy between Percutaneous Coronary Intervention with TAXUS and Cardiac Surgery.

Model/Outcome	Predictor	OR (95% CI)	*p*
LDL-C <1.4 mmol/L at 12 months	Statin discontinued/irregular use	0.07 (0.05–0.11)	<0.001
LDL-C <1.4 mmol/L at 12 months	Ezetimibe-containing discharge therapy	3.24 (1.67–6.28)	<0.001
LDL-C <1.4 mmol/L at 12 months	Non-statin process-of-care measure, per point	1.32 (1.18–1.47)	<0.001
LDL-C <1.4 mmol/L at 12 months	Admission LDL-C, per 1 mmol/L	0.54 (0.48–0.60)	<0.001
One-year death	Age, per 10 years	1.82 (1.53–2.17)	<0.001
One-year death	LVEF <40%	4.81 (3.42–6.78)	<0.001
One-year death	SYNTAX score, per 10 points	1.33 (1.15–1.54)	<0.001
One-year death	CRP above median	2.30 (1.63–3.25)	<0.001
One-year ischemic MACE	LVEF <40%	2.51 (1.86–3.39)	<0.001
One-year ischemic MACE	SYNTAX score, per 10 points	1.52 (1.35–1.71)	<0.001
One-year ischemic MACE	CRP above median	1.50 (1.16–1.95)	0.002
One-year broad MACE	Diabetes mellitus	1.42 (1.10–1.84)	0.007
One-year broad MACE	LVEF <40%	2.46 (1.86–3.27)	<0.001
One-year broad MACE	SYNTAX score, per 10 points	1.39 (1.24–1.55)	<0.001
One-year broad MACE	CRP above median	1.44 (1.14–1.82)	0.002

## Data Availability

De-identified aggregate outputs are available from the corresponding author upon reasonable request and institutional approval. Individual patient-level data are not publicly available because of institutional and ethical restrictions.

## Supplementary Materials

The following supporting information can be downloaded at: https://www.mdpi.com/article/10.3390/medicina62071342/s1, Supplementary Tables S1–S9, including outcome definitions, characterization of missing lifestyle data, MINOCA subset, statin non-persistence reasons, lipid-lowering treatment escalation, anthropometric and glycemic data, LDL-C target model, one-year outcome models and inflammatory/neurohormonal markers.

## References

[1] Byrne R.A., Rossello X., Coughlan J.J., Barbato E., Berry C., Chieffo A., Claeys M.J., Dan G.-A., Dweck M.R., Galbraith M. (2023). 2023 ESC Guidelines for the management of acute coronary syndromes. Eur. Heart J..

[2] Mach F., Baigent C., Catapano A.L., Koskinas K.C., Casula M., Badimon L., Chapman M.J., De Backer G.G., Delgado V., Ference B.A. (2020). 2019 ESC/EAS Guidelines for the management of dyslipidaemias. Eur. Heart J..

[3] Visseren F.L.J., Mach F., Smulders Y.M., Carballo D., Koskinas K.C., Bäck M., Benetos A., Biffi A., Boavida J.-M., Capodanno D. (2021). 2021 ESC Guidelines on cardiovascular disease prevention in clinical practice. Eur. Heart J..

[4] Piepoli M.F., Corrà U., Adamopoulos S., Benzer W., Bjarnason-Wehrens B., Cupples M., Dendale P., Doherty P., Gaita D., Höfer S. (2014). Secondary prevention in the clinical management of patients with cardiovascular diseases: Core components, standards and outcome measures for referral and delivery. Eur. J. Prev. Cardiol..

[5] Kotseva K., De Backer G., De Bacquer D., Rydén L., Hoes A., Grobbee D., Maggioni A., Marques-Vidal P., Jennings C., Abreu A. (2019). Lifestyle and impact on cardiovascular risk factor control in coronary patients across 27 countries: EUROASPIRE V. Eur. J. Prev. Cardiol..

[6] Ray K.K., Haq I., Bilitou A., Manu M.C., Burden A., Aguiar C., Arca M., Connolly D.L., Eriksson M., Ferrières J. (2023). Treatment gaps in the implementation of LDL cholesterol control among high- and very high-risk patients in Europe between 2020 and 2021: The multinational observational SANTORINI study. Lancet Reg. Health Eur..

[7] Bäck M., Leosdottir M., Hagström E., Norhammar A., Hag E., Jernberg T., Wallentin L., Lindahl B., Hambraeus K. (2021). SWEDEHEART Study Group. The SWEDEHEART secondary prevention and cardiac rehabilitation registry (SWEDEHEART CR registry). Eur. Heart J. Qual. Care Clin. Outcomes.

[8] Brown R., Lewsey J., Wild S., Logue J., Welsh P. (2021). Associations of statin adherence and lipid targets with adverse outcomes in myocardial infarction survivors: A retrospective cohort study. BMJ Open.

[9] Puymirat E., Bonaca M., Iliou M.C., Tea V., Ducrocq G., Douard H., Labrunee M., Plastaras P., Chevallereau P., Taldir G. (2019). Outcome associated with prescription of cardiac rehabilitation according to predicted risk after acute myocardial infarction: Insights from the FAST-MI registries. Arch. Cardiovasc. Dis..

[10] Thygesen K., Alpert J.S., Jaffe A.S., Chaitman B.R., Bax J.J., Morrow D.A., White H.D., ESC Scientific Document Group (2019). Fourth universal definition of myocardial infarction. Eur. Heart J..

[11] Sianos G., Morel M.-A., Kappetein A.P., Morice M.-C., Colombo A., Dawkins K., Brand M.V.D., Van Dyck N., Russell M.E., Mohr F.W. (2005). The SYNTAX Score: An angiographic tool grading the complexity of coronary artery disease. EuroIntervention.

[12] Martínez-González M.A., García-Arellano A., Toledo E., Salas-Salvadó J., Buil-Cosiales P., Corella D., Covas M.I., Schröder H., Arós F., Gómez-Gracia E. (2012). A 14-item Mediterranean diet assessment tool and obesity indexes among high-risk subjects: The PREDIMED trial. PLoS ONE.

[13] Schröder H., Fitó M., Estruch R., Martínez-González M.A., Corella D., Salas-Salvadó J., Lamuela-Raventós R., Ros E., Salaverría I., Fiol M. (2011). A short screener is valid for assessing Mediterranean diet adherence among older Spanish men and women. J. Nutr..

[14] Delgado-Lista J., Alcala-Diaz J.F., Torres-Peña J.D., Quintana-Navarro G.M., Fuentes F., Garcia-Rios A., Ortiz-Morales A.M., Perez-Caballero A.I., Yubero-Serrano E.M., Rangel-Zuñiga O.A. (2022). Long-term secondary prevention of cardiovascular disease with a Mediterranean diet and a low-fat diet (CORDIOPREV): A randomised controlled trial. Lancet.

[15] Von Elm E., Altman D.G., Egger M., Pocock S.J., Gøtzsche P.C., Vandenbroucke J.P., Initiative S. (2007). The Strengthening the Reporting of Observational Studies in Epidemiology (STROBE) statement: Guidelines for reporting observational studies. Lancet.

[16] Pedretti R.F.E., Hansen D., Ambrosetti M., Back M., Berger T., Ferreira M.C., Cornelissen V., Davos C.H., Doehner W., Zarzosa C.d.P.Y. (2023). How to optimize the adherence to guideline-directed medical therapy in the secondary prevention of cardiovascular diseases: A clinical consensus statement from the European Association of Preventive Cardiology. Eur. J. Prev. Cardiol..

[17] Sabatine M.S., Giugliano R.P., Keech A.C., Honarpour N., Wiviott S.D., Murphy S.A., Kuder J.F., Wang H., Liu T., Wasserman S.M. (2017). Evolocumab and clinical outcomes in patients with cardiovascular disease. N. Engl. J. Med..

[18] Schwartz G.G., Steg P.G., Szarek M., Bhatt D.L., Bittner V.A., Diaz R., Edelberg J.M., Goodman S.G., Hanotin C., Harrington R.A. (2018). Alirocumab and cardiovascular outcomes after acute coronary syndrome. N. Engl. J. Med..

[19] Anderson L., Oldridge N., Thompson D.R., Zwisler A.-D., Rees K., Martin N., Taylor R.S. (2016). Exercise-based cardiac rehabilitation for coronary heart disease. J. Am. Coll. Cardiol..

[20] Critchley J.A., Capewell S. (2003). Mortality risk reduction associated with smoking cessation in patients with coronary heart disease: A systematic review. JAMA.

