# Supplementary material for: One-Year Gaps in Comprehensive Secondary Prevention After Acute Myocardial Infarction: Statin Persistence, LDL-C Target Achievement, Rehabilitation, and Lifestyle Adherence
Source: Medicina (Kaunas). 2026 Jul 12;62(7):1342. doi: 10.3390/medicina62071342 (PMC13413717; doi:10.3390/medicina62071342)
Supplement: Supplementary file 1 [file medicina-62-01342-s001.zip › medicina-4414325-supplementary.pdf]

Supplementary Materials

Article title: One-Year Gaps in Comprehensive Secondary Prevention After Acute Myocardial Infarction: Statin Persistence, LDL-C Target Achievement, Rehabilitation, and Lifestyle Adherence

Corresponding author: Ivana Jurin, MD; Department of Cardiology, Dubrava University Hospital, Zagreb, Croatia; e-mail: ivanajurin1912@gmail.com

Abbreviations: AMI, acute myocardial infarction; BMI, body mass index; CI, confidence interval; CRP, C-reactive protein; HbA1c, glycated hemoglobin; GLP-1, glucagon-like peptide-1; PCSK9, proprotein convertase subtilisin/kexin type 9; SGLT2, sodium-glucose cotransporter 2; LDL-C, low-density lipoprotein cholesterol; LVEF, left ventricular ejection fraction; MACE, major adverse cardiovascular events; MINOCA, myocardial infarction with non-obstructive coronary arteries; NT-proBNP, N-terminal pro-B-type natriuretic peptide; OR, odds ratio; STEMI, ST-elevation myocardial infarction; SYNTAX, Synergy between Percutaneous Coronary Intervention with TAXUS and Cardiac Surgery.

Supplementary Table S1. One-year outcome definitions and evaluability rules.

Variable/outcome	Definition used in analysis	One-year evaluability rule
One-year death	All-cause death within 365 days after index AMI	Death within 365 days or at least 365 event-free follow-up days
Cardiovascular death	Cause recorded as sudden death, myocardial infarction, heart failure, stroke or pulmonary embolism	Same as mortality evaluability
Ischemic MACE	Cardiovascular death, recurrent myocardial infarction, stroke/TIA, unplanned coronary revascularization, stent restenosis/occlusion or stent thrombosis	Event within 365 days or at least 365 event-free follow-up days
Broad MACE	Ischemic MACE plus heart failure events, clinically relevant bleeding, pulmonary embolism or venous thromboembolism when recorded as major follow-up events	Event within 365 days or at least 365 event-free follow-up days
Stent thrombosis	Definite/probable registry-recorded stent thrombosis within 365 days	Event within 365 days or at least 365 event-free follow-up days

Supplementary Table S2. Comparison of patients with complete versus incomplete observed lifestyle data. Complete observed lifestyle data were defined as observed regular exercise, dietary pattern and follow-up smoking status. Values are median [interquartile range] or n/N (%).

Variable	Complete observed lifestyle data	Incomplete observed lifestyle data	p
Patients	2002	974	-
Age, years	63.0 [55.0-71.0]	68.0 [60.0-77.0]	<0.001
Women	581/2002 (29.0)	341/974 (35.0)	0.001
STEMI	1115/2002 (55.7)	533/974 (54.7)	0.645
Diabetes mellitus	449/2002 (22.4)	279/974 (28.6)	<0.001
LVEF, %	55.0 [45.0-60.0]	50.0 [40.0-55.0]	<0.001
SYNTAX score	13.0 [8.0-20.5]	17.5 [9.0-25.0]	<0.001
One-year death	7/1988 (0.4)	281/954 (29.5)	<0.001
One-year ischemic MACE	120/1987 (6.0)	275/915 (30.1)	<0.001
One-year broad MACE	169/1987 (8.5)	309/915 (33.8)	<0.001

Supplementary Table S3. MINOCA subset and observed secondary prevention pathway data. MINOCA, myocardial infarction with non-obstructive coronary arteries.

Variable	MINOCA subset
MINOCA patients	6/2976 (0.2)
Statin at discharge	6/6 (100.0)
Statin discontinued/irregular use	4/6 (66.7)
LDL-C <1.4 mmol/L	0/2 (0.0)
Rehabilitation	0/6 (0.0)
Regular exercise	1/6 (16.7)
Favourable dietary pattern	1/4 (25.0)
One-year death	2/6 (33.3)
One-year ischemic MACE	2/6 (33.3)

Supplementary Table S4. Recorded statin discontinuation/irregular-use categories and reasons. Data are patient-reported and registry-recorded when available. Adverse effects are shown as the primary recorded reason category; any reported statin side effects are summarized separately.

Category	Result
Completely discontinued	288/2634 (10.9)
Irregular use	626/2634 (23.8)
Lipids perceived as good	189/713 (26.5)
Negative beliefs about statins	37/713 (5.2)
Reported taking therapy	167/713 (23.4)
Not informed that therapy was needed	45/713 (6.3)
Adverse effects as primary recorded reason	73/713 (10.2)
Forgetfulness/does not know	114/713 (16.0)
Unknown reason	14/713 (2.0)
Perceived not needed	65/713 (9.1)
Any reported statin side effects among non-persistent/irregular users	179/714 (25.1)

Supplementary Table S5. Descriptive lipid-lowering treatment escalation information recorded in the registry. Unless otherwise stated, denominators use the full analytic cohort. These data were not captured as a uniform prespecified endpoint throughout the entire study period.

Therapeutic escalation descriptor	Result
Ezetimibe-containing discharge regimen	389/2976 (13.1)
Any recorded follow-up ezetimibe-containing regimen or addition	548/2976 (18.4)
Any recorded PCSK9/inclisiran/bempedoic acid use, addition or recommendation	59/2976 (2.0)
Any recorded follow-up ezetimibe-containing regimen/addition among LDL-C non-target patients	419/1817 (23.1)
Any recorded PCSK9/inclisiran/bempedoic acid use/addition/recommendation among LDL-C non-target patients	40/1817 (2.2)

Supplementary Table S6. Anthropometric, glycemic and contemporary glucose-lowering therapy data available in the registry.

Variable	Result
BMI at index hospitalization	28.4 [25.6-31.4] (n=2853)
Obesity, BMI >=30 kg/m2	1063/2853 (37.3)
BMI follow-up available	1966/2976 (66.1)
Paired BMI change (follow-up minus baseline)	-0.2 [-1.4-0.6] (n=1962)
Baseline HbA1c	6.1 [5.7-7.1] (n=1682)
Follow-up HbA1c	7.0 [6.3-8.0] (n=564)
GLP-1 receptor agonist/tirzepatide at discharge	119/2835 (4.2)
GLP-1 receptor agonist/tirzepatide at follow-up	137/2032 (6.7)
SGLT2 inhibitor at discharge	609/2835 (21.5)
SGLT2 inhibitor at follow-up	441/2033 (21.7)

Supplementary Table S7. Adjusted logistic regression model for LDL-C <1.4 mmol/L at 12 months. The non-statin process-of-care measure excludes statin persistence and is interpreted as a descriptive process measure rather than a validated risk score.

Predictor	N	LDL-C target events	OR (95% CI)	p
Statin discontinued/irregular use	1935	529	0.07 (0.05-0.11)	<0.001
Ezetimibe-containing discharge therapy	1935	529	3.24 (1.67-6.28)	<0.001
Non-statin process-of-care measure, per point	1935	529	1.32 (1.18-1.47)	<0.001
Admission LDL-C, per 1 mmol/L	1935	529	0.54 (0.48-0.60)	<0.001
Female sex	1935	529	0.95 (0.73-1.23)	0.689
Diabetes mellitus	1935	529	0.96 (0.72-1.28)	0.789
Age, per 10 years	1935	529	0.93 (0.84-1.03)	0.169

Supplementary Table S8. Full baseline/severity-adjusted one-year outcome models. Post-discharge rehabilitation and follow-up smoking status were not included to avoid immortal-time and healthy-user bias.

Outcome	Predictor	N	Events	OR (95% CI)	p
One-year death	Age, per 10 years	2530	199	1.82 (1.53-2.17)	<0.001
One-year death	Female sex	2530	199	1.24 (0.88-1.74)	0.223
One-year death	Diabetes mellitus	2530	199	1.24 (0.88-1.75)	0.222
One-year death	ST-elevation myocardial infarction	2530	199	1.40 (1.00-1.96)	0.047
One-year death	Prior myocardial infarction	2530	199	0.93 (0.57-1.51)	0.759
One-year death	LVEF <40%	2530	199	4.81 (3.42-6.78)	<0.001
One-year death	SYNTAX score, per 10 points	2530	199	1.33 (1.15-1.54)	<0.001
One-year death	Active smoking at AMI	2530	199	0.88 (0.61-1.26)	0.475
One-year death	CRP above median	2530	199	2.30 (1.63-3.25)	<0.001
One-year ischemic MACE	Age, per 10 years	2500	303	1.34 (1.18-1.53)	<0.001
One-year ischemic MACE	Female sex	2500	303	1.13 (0.85-1.49)	0.410
One-year ischemic MACE	Diabetes mellitus	2500	303	1.31 (0.98-1.73)	0.064
One-year ischemic MACE	ST-elevation myocardial infarction	2500	303	1.38 (1.05-1.80)	0.019
One-year ischemic MACE	Prior myocardial infarction	2500	303	1.28 (0.88-1.85)	0.193
One-year ischemic MACE	LVEF <40%	2500	303	2.51 (1.86-3.39)	<0.001
One-year ischemic MACE	SYNTAX score, per 10 points	2500	303	1.52 (1.35-1.71)	<0.001
One-year ischemic MACE	Active smoking at AMI	2500	303	1.01 (0.76-1.33)	0.949
One-year ischemic MACE	CRP above median	2500	303	1.50 (1.16-1.95)	0.002
One-year broad MACE	Age, per 10 years	2500	382	1.36 (1.21-1.53)	<0.001
One-year broad MACE	Female sex	2500	382	1.17 (0.91-1.51)	0.218
One-year broad MACE	Diabetes mellitus	2500	382	1.42 (1.10-1.84)	0.007
One-year broad MACE	ST-elevation myocardial infarction	2500	382	1.27 (1.00-1.62)	0.049
One-year broad MACE	Prior myocardial infarction	2500	382	1.10 (0.78-1.55)	0.591
One-year broad MACE	LVEF <40%	2500	382	2.46 (1.86-3.27)	<0.001
One-year broad MACE	SYNTAX score, per 10 points	2500	382	1.39 (1.24-1.55)	<0.001
One-year broad MACE	Active smoking at AMI	2500	382	1.18 (0.92-1.52)	0.197
One-year broad MACE	CRP above median	2500	382	1.44 (1.14-1.82)	0.002

Supplementary Table S9. Inflammatory and neurohormonal markers by one-year death and ischemic MACE. Values are median [interquartile range] (n).

Marker	Outcome	No event	Event	p
C-reactive protein, mg/L	One-year death	4.3 [1.9-9.4] (n=2467)	12.5 [3.3-61.4] (n=272)	<0.001
C-reactive protein, mg/L	One-year ischemic MACE	4.3 [1.9-9.4] (n=2324)	6.9 [2.4-27.9] (n=379)	<0.001
Fibrinogen, g/L	One-year death	3.8 [3.3-4.4] (n=659)	4.2 [3.5-5.5] (n=93)	<0.001
Fibrinogen, g/L	One-year ischemic MACE	3.8 [3.3-4.4] (n=619)	4.2 [3.4-5.0] (n=125)	0.002
Albumin, g/L	One-year death	40.0 [38.0-43.0] (n=976)	34.0 [31.8-38.0] (n=140)	<0.001
Albumin, g/L	One-year ischemic MACE	40.0 [38.0-43.0] (n=924)	36.0 [32.2-39.0] (n=174)	<0.001
NT-proBNP, pg/mL	One-year death	885.5 [273.8-2647.3] (n=1032)	5191.5 [1660.3-11536.8] (n=118)	<0.001
NT-proBNP, pg/mL	One-year ischemic MACE	870.0 [268.5-2598.8] (n=972)	3207.0 [988.0-8802.0] (n=157)	<0.001
Neutrophil-to-lymphocyte ratio	One-year death	3.5 [2.2-5.8] (n=2515)	5.3 [3.0-9.7] (n=274)	<0.001
Neutrophil-to-lymphocyte ratio	One-year ischemic MACE	3.5 [2.2-5.8] (n=2371)	4.6 [2.4-8.4] (n=380)	<0.001
